# Elevated Expression of Programmed Death-1 and Programmed Death Ligand-1 Negatively Regulates Immune Response against Cervical Cancer Cells

**DOI:** 10.1155/2016/6891482

**Published:** 2016-09-05

**Authors:** Zhifang Chen, Nannan Pang, Rong Du, Yuejie Zhu, Lingling Fan, Donghui Cai, Yan Ding, Jianbing Ding

**Affiliations:** ^1^Department of Gynecology, The First Affiliated Hospital of Xinjiang Medical University, Urumqi 830054, China; ^2^Department of Hematology, The First Affiliated Hospital of Xinjiang Medical University, Urumqi 830054, China; ^3^Department of Immunology, Xinjiang Medical University, Urumqi 830011, China

## Abstract

The present study is to measure the expression of programmed death-1 (PD-1) and programmed death ligand-1 (PD-L1), as well as its clinical significance in cervical cancer patients. Our results showed that different T cell subsets in patients with cervical cancer had high expression of PD-1, and DCs had high expression of PD-L1. High expression of PD-1 on Treg cells in cervical cancer patients facilitated the production of TGF-*β* and IL-10 but inhibited the production of IFN-*γ*. Cervical cancer elevated the expression of PD-1 and PD-L1 in mRNA level. PD-1 expression in peripheral blood of cervical cancer patients was related with tumor differentiation, lymph node metastasis, and invasiveness. PD-1/PD-L1 pathway inhibited lymphocyte proliferation but enhanced the secretion of IL-10 and TGF-*β* in vitro. In summary, our findings demonstrate that elevated levels of PD-1/PD-L1, TGF-*β*, and IL-10 in peripheral blood of cervical cancer patients may negatively regulate immune response against cervical cancer cells and contribute to the progression of cervical cancer. Therefore, PD-1/PD-L1 pathway may become an immunotherapy target in the future.

## 1. Introduction

Cervical cancer has the second highest morbidity and mortality rates among all female genital tract malignant tumors [1]. Surgery, radiotherapy, and/or interventional chemotherapy are the most commonly used treatment methods for cervical cancer, but their effects are still limited. As the development of tumor immunology and molecular biology, biological treatment has become the fourth treatment method for malignant tumors [2]. The incidence of cervical cancer in Xinjiang Uygur women is significantly higher than that in women of other ethnic groups, with an average age of onset at 43 years old [3]. In Xinjiang Autonomous Region, cervical cancer severely threatens the health and life quality of Uygur women and has become an important public health problem [4].

Programmed death-l (PD-1) is a recently discovered costimulatory molecule on the surface of T cells that binds with PD-ligand to conduct inhibitory second signals. Programmed death ligand-1 (PD-L1) is a member of costimulatory signal B7 family and plays an important role in tumor immune responses [5]. PD-L1 is widely expressed in various types of immune cells, epithelial cells, and human tumor cells [5]. PD-1, an immune inhibitory receptor that is expressed during lymphocyte activation process, is the receptor of PD-L1. No existence of PD-1 molecules is found in resting T cells or B cells [6]. It has been demonstrated that PD-L1 is highly expressed in human tumor cells and antigen-presenting cells in tumor microenvironments. PD-L1 interacts with PD-1 on the surface of T cells, inhibits the activation of tumor antigen-specific T cells, and induces immune tolerance of T cells to tumor cells. This might be one of the important mechanisms by which tumor cells evade immune surveillance [5–7]. In the present study, we detect PD-1 and PD-L1, as well as related cytokines in cervical lesion development process, and analyze the relevance between changes in PD-1/PD-L1 and CD4+ T cells, CD8+ T cells, and Treg cells to evaluate whether PD-1/PD-L1 can be used as an early clinical warning indicator of cervical cancer.

## 2. Materials and Methods

### 2.1. Patients

A total of 78 patients (age range, 36–60 years; average age, 45.50 ± 6.12 years) who were first diagnosed with cervical cancer by colposcopy pathological examination at our hospital between January 2010 and October 2014 were included in the present study (cervical cancer group) (Table 1). Another group of 53 untreated patients with cervical intraepithelial neoplasia (CIN) by colposcopy pathological examination aged 26–58 years (average, 44.24 ± 5.67 years) were included as CIN group (Table 2). In addition, 40 healthy volunteer subjects aged 24–66 years (average, 45.35 ± 6.17 years) were included as normal control group. There was no statistically significant difference in age among the three groups. Exclusion criteria included (i) history of diabetes, (ii) hypertension, (iii) cardiovascular disease, (iv) pregnancy, and (v) acute or chronic infectious disease or metastatic tumor. Histological and clinical staging of all cervical cancer patients was performed according to FIGO staging standards published in 2000. All procedures were approved by the Ethics Committee of Xinjiang Medical University. Written informed consents were obtained from all patients or their families.

### 2.2. Flow Cytometry

Peripheral blood mononuclear cells (PBMCs) were separated from 5 mL venous blood using Ficoll-Hypaque density gradient centrifugation. For the detection of PD-1, two aliquots of PBMCs (20 *μ*L) were mixed with APC-CD4, PECy7-CD25, PE-PD-1, and FITC-CD8 antibodies (20 *μ*L each) and APC-CD4, PECy7-CD25, PE-IgG, and FITC-CD8 antibodies (20 *μ*L each), respectively. Then, the mixtures were shaken before incubation at room temperature for 15 minutes in the dark. Afterwards, the mixtures were washed with phosphate-buffered saline (PBS) twice before adding 400 *μ*L PBS to resuspend the cells. For the detection of PD-L1, one aliquot of PBMCs (20 *μ*L) was mixed with PERCP-CD11c and PE-PD-L1 antibodies (20 *μ*L each), and the other aliquot of PBMCs (20 *μ*L) was mixed with PERCP-CD11c and PE-IgG (20 *μ*L each). After shaking, the mixtures were incubated at room temperature for 15 minutes in the dark before washing with PBS twice. Then, 400 *μ*L PBS was added to resuspend the cells. After the samples were prepared, they were subjected to flow cytometry using FACS Aria II (Becton Dickinson, Franklin Lakes, NJ, USA) for the detection of PD-1 expression on CD4+ and CD8+T cell surface and PD-L1 expression on dendritic cell (DC) surface.

### 2.3. Enzyme-Linked Immunosorbent Assay (ELISA)

The contents of transforming growth factor- (TGF-) *β*, interleukin- (IL-) 10, and interferon- (IFN-) *γ* in serum were determined using ELISA kits (Bender MedSystems, Vienna, Austria) according to the manufacturer's manuals. The optical density at 450 nm was measured using a reader (ELx800; BioTek, Winooski, VT, USA).

### 2.4. Quantitative Real-Time Polymerase Chain Reaction (qRT-PCR)

Peripheral blood (5 mL) was collected from fasting healthy subjects and patients in the morning and plasma was extracted. Total RNA was extracted from PBMC of three groups using TRIzol (Thermo Fisher Scientific, Waltham, MA, USA). Synthesis of cDNA first strand was performed using Fermentas kit (Thermo Fisher Scientific, Waltham, MA, USA) according to the manufacturer's protocols. The sequences of primers for PD-1 (289 bp) were TGCAGCTTCTCCAACACATC (upstream) and CTGCCCTTCTCTCTGTCACC (downstream). The sequences of primers for PD-L1 (101 bp) were CCTGGAGGTTTCGAGATTCA (upstream) and GGCAAAGCCAAGGTACTCC (downstream). The sequences of primers for *β*-actin (233 bp) were TAGGCGGACTGTTACTGAGC (upstream) and TGCTCCAACCAACTGCTGTC (downstream). The PCR reaction system (20 *μ*L) was made using SYBR-Premix Ex TaqTM II according to the manufacturer's manual (Takara, Tokyo, Japan). PCR reaction conditions were denaturation at 95°C for 30 s, followed by 40 cycles of 95°C for 5 s and 60°C for 30 s. The data were analyzed using iQ520 Standard Edition Optical System Software V2.0 (Bio-Rad, Hercules, CA, USA). *β*-actin was used as internal control. The 2^−ΔΔCt^ method was used to determine the cycle number Ct value corresponding to a specific fluorescence threshold and to quantify the target genes. Experiments were repeated for at least 3 times. The level of mRNA was expressed as the ratio of target gene against *β*-actin.

### 2.5. MTT Assay and Cytometric Bead Array (CBA) Assay

Lymphocytes and DCs separated from cervical tissues were labeled with anti-CD3 antibody (Becton Dickinson, Franklin Lakes, NJ, USA) and anti-CD11c antibody (Invitrogen, Thermo Fisher Scientific, Waltham, MA, USA). Lymphocytes (CD3+) and DCs (CD11c+) were separated using MACS system (Miltenyi Biotec, Bergisch Gladbach, Germany). The density of cells was adjusted to 1 × 10^6^ cells/mL using RPMI-1640 medium. Then, 100 *μ*L cell suspension was added into 96-well culture plates (1 × 10^5^ cells/well) for cultivation of 72 h. To block PD-1/PD-L1 pathway, anti-PD-L1 antibody (final concentration of 2 *μ*g/mL) was added before incubation for 72 h. The cells were divided into normal control group, control + anti-PD-L1 antibody group, CIN group, CIN + anti-PD-L1 antibody group, cervical cancer group, and cervical cancer + anti-PD-L1 antibody group. Stimulator ConA was added into the medium (final concentration of 3 *μ*g/mL), followed by incubation at 37°C in 5% CO_2_ for 72 h. MTT assay was performed to analyze lymphocyte proliferation. Culture supernatants from each group were subjected to CBA assay for the determination of IL-10 and TGF-*β* levels.

### 2.6. Statistical Analysis

All results were analyzed using SPSS 16.0 statistical software (IBM, Armonk, NY, USA). The data were expressed as means ± standard deviations. Intergroup comparison of ages was performed using *t*-test. Differences between groups were compared using analysis of variance. Comparison among groups was performed using *χ* ^2^ test.

## 3. Results

### 3.1. Different T Cell Subsets in Patients with Cervical Cancer Have High Expression of PD-1, and DCs Have High Expression of PD-L1

**Figure 1:**
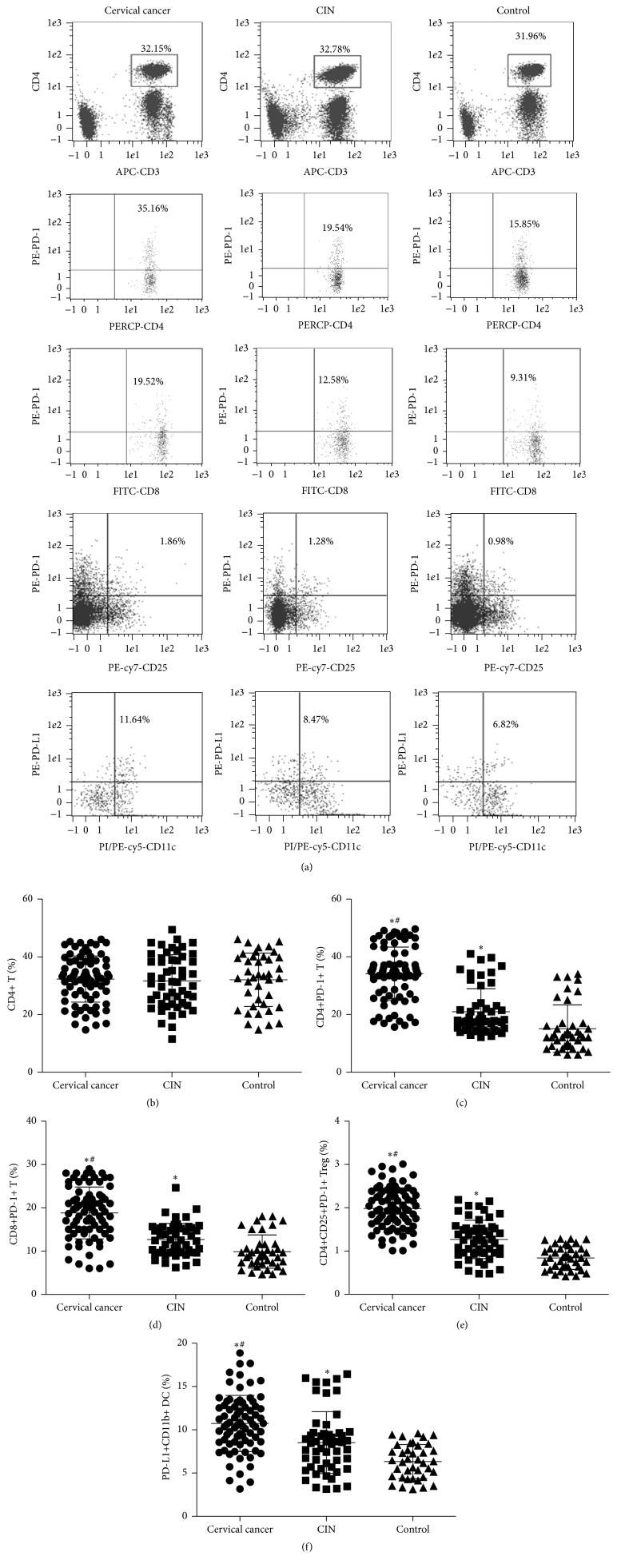
PD-1 expression in T cells and PD-L1 expression in DCs. (a) Representative flow cytometric plots for the measurements of the contents of (b) CD4+T, (c) CD4+ PD-1+T, (d) CD8+ PD-1+T, (e) CD4+CD25+PD-1+Treg, and (f) PD-L1+CD11b+DC in normal control, CIN, and cervical cancer groups. ^*∗*^ *P* < 0.05 compared with control group; ^#^ *P* < 0.05 compared with CIN group.

To measure PD-1 expression in T cells and PD-L1 expression in DCs, flow cytometry was employed. The data showed that the percentage of CD4+ T cells was not significantly different among normal control group, CIN group, and cervical cancer group (*P* > 0.05) (Figures 1(a) and 1(b)). The percentages of CD4+PD-1+ T cells, CD8+PD-1+ T cells, or CD4+CD25+PD-1+ Treg cells were significantly different among normal control group, CIN group, and cervical cancer group (*P* < 0.05 for all) (Figures 1(a)–1(e)). In addition, the percentage of PD-L1 + DCs was significantly different among the three groups (*P* < 0.05) (Figures 1(a) and 1(f)). These results suggest that different T cell subsets in patients with cervical cancer have high expression of PD-1, and DCs have high expression of PD-L1.

### 3.2. High Expression of PD-1 on Treg Cells in Cervical Cancer Patients Facilitates the Production of TGF-*β* and IL-10 but Inhibits the Production of IFN-*γ*

**Figure 2:**
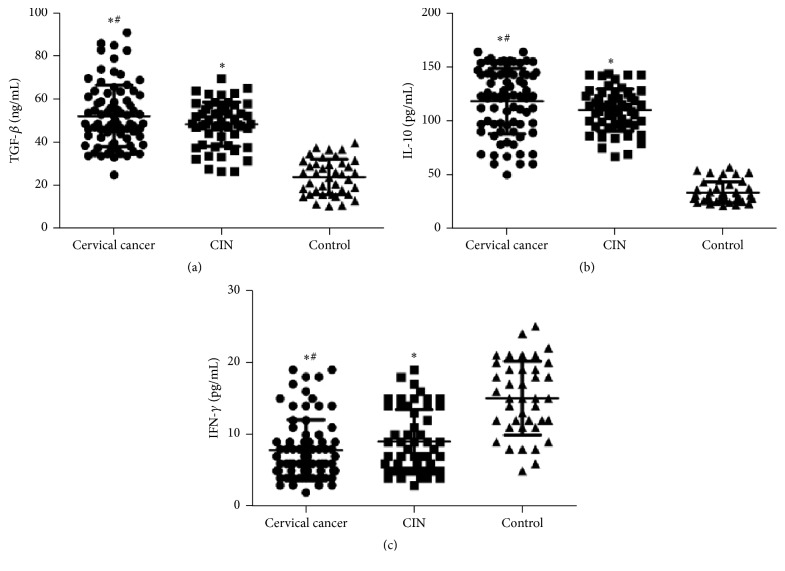
Contents of cytokines related with PD-1 and PD-L1. ELISA was performed to measure the levels of TGF-*β*, IL-10, and IFN-*γ* in normal control, CIN, and cervical cancer groups. ^*∗*^ *P* < 0.05 compared with control group; ^#^ *P* < 0.05 compared with CIN group.

**Figure 3:**
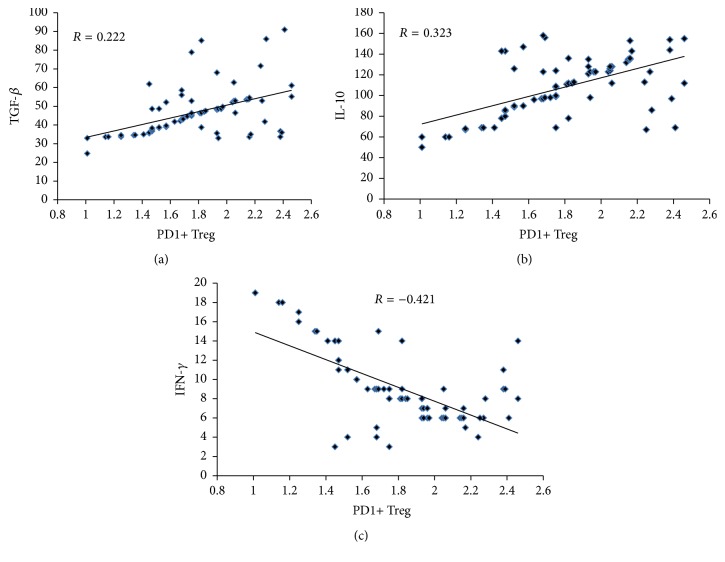
Correlation analyses between CD4+CD25+PD-1+Treg and (a) TGF-*β*, (b) IL-10, or (c) IFN-*γ*. The fitted lines in (a) and (b) indicate positive correlation, while the fitted line in (c) shows negative correlation.

To determine the contents of cytokines related with PD-1 and PD-L1, ELISA and correlation analysis were performed. The data showed that the levels of TGF-*β*, IL-10, and IFN-*γ* were significantly different among cervical cancer group, CIN group, and control group (*P* < 0.05). In cervical cancer patients, the levels of TGF-*β* and IL-10 were significantly enhanced, and the level of IFN-*γ* was significantly reduced (Figure 2). Correlation analyses between CD4+CD25+PD-1+Treg and TGF-*β*, IFN-*γ* or IL-10 showed that CD4+CD25+PD-1+Treg was positively correlated with TGF-*β* and IL-10 (*r* = 0.222 and 0.323, resp.) and was negatively correlated with IFN-*γ* (*r* = −0.421) (Figure 3). These results indicate that high expression of PD-1 on Treg cells in cervical cancer patients facilitates the production of TGF-*β* and IL-10 but inhibits the production of IFN-*γ*.

### 3.3. Cervical Cancer Elevates the Expression of PD-1 and PD-L1 in mRNA Level

**Figure 4:**
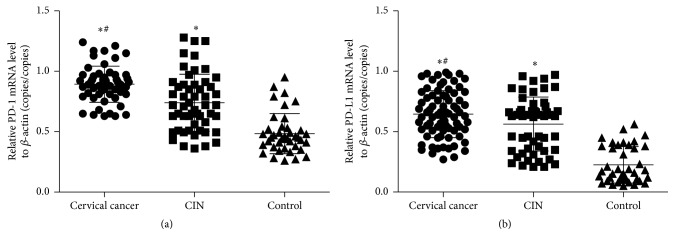
The mRNA expression levels of PD-1 and PD-L1. qRT-PCR was used to measure (a) PD-1 mRNA level and (b) PD-L1 mRNA level in normal control, CIN, and cervical cancer groups. ^*∗*^ *P* < 0.05 compared with control group; ^#^ *P* < 0.05 compared with CIN group.

To measure mRNA expression of PD-1 and PD-L1, QRT-PCR was used. The data showed that the mRNA levels of PD-1 and PD-L1 were both increased as the disease condition was aggravated, with the mRNA levels in cervical cancer group being significantly higher than those of control groups (*P* < 0.05) (Figure 4). The result suggests that cervical cancer elevates the expression of PD-1 and PD-L1 in mRNA level.

### 3.4. PD-1 Expression on CD8+T of Cervical Cancer Patients Is Related with Tumor Differentiation, Lymph Node Metastasis, and Invasiveness

**Figure 5:**
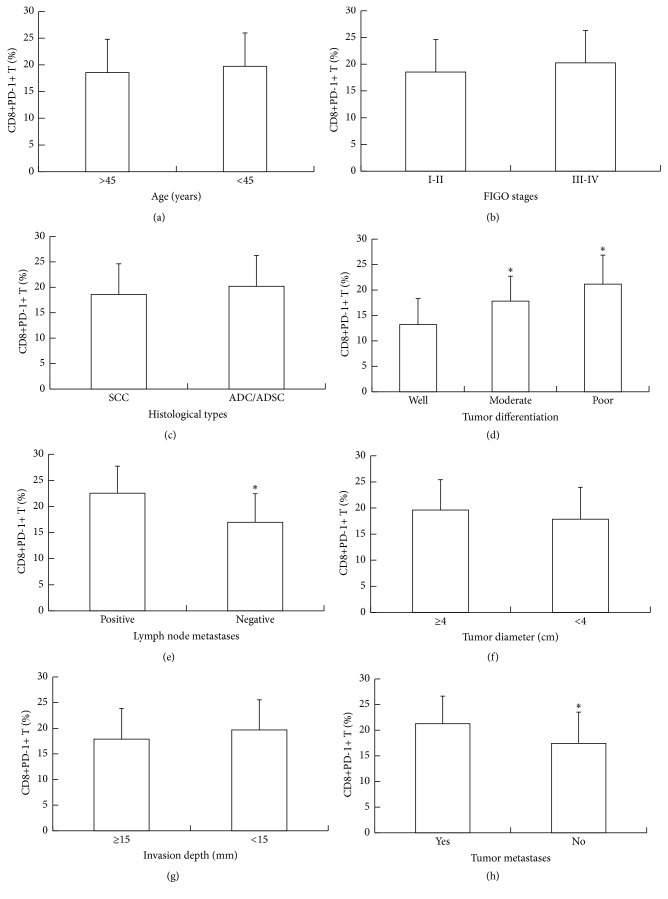
Relationship between PD-1 expression on CD8+T of cervical cancer patients and (a) age, (b) tumor staging, (c) histological types, (d) tumor differentiation, (e) lymph node metastasis, (f) tumor diameter, (g) invasion depth, and (h) tumor metastasis. ^*∗*^ *P* < 0.05 compared with the group without “*∗*.”

To further test how PD-1 expression in peripheral blood affects clinical characteristics based on the expression of PD-1^+^ on CD8+T cells, we studied clinical characteristics such as age, tumor staging, histological types, tumor differentiation, lymph node metastasis, tumor diameter, invasion depth, and tumor metastasis. The data showed that the expression of PD-1 in CD8+T was related with tumor differentiation, lymph node metastasis, and tumor metastasis (*P* < 0.05), but not age, tumor staging, histological types, tumor diameter, or invasion depth (*P* > 0.05) (Figure 5). The result indicates that PD-1 expression on CD8+T is related with cervical cancer differentiation, lymph node metastasis, and tumor metastasis.

### 3.5. PD-1/PD-L1 Pathway Inhibits Lymphocyte Proliferation but Enhances the Secretion of IL-10 and TGF-*β* In Vitro

**Figure 6:**
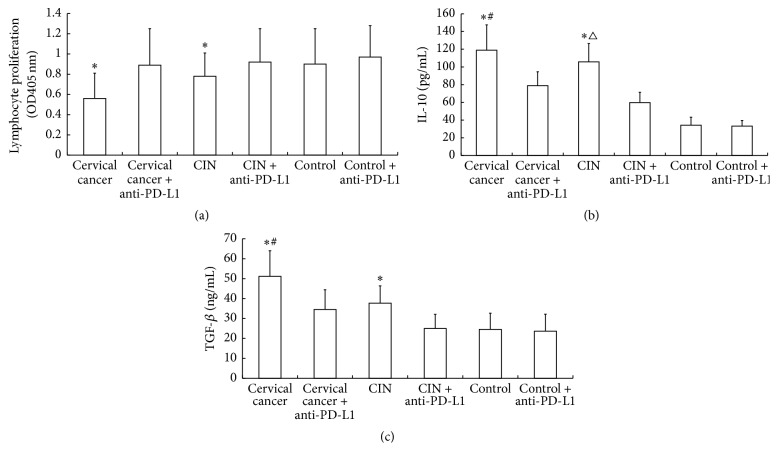
Effect of PD-L1 on lymphocyte proliferation and the secretion of IL-10 and TGF-*β*. Anti-PD-L1 antibody was used to block PD-L1. (a) Lymphocyte proliferation determined using MTT assay. Concentrations of (b) IL-10 and (c) TGF-*β* of culture supernatants measured using CBA assay. ^*∗*^ *P* < 0.05 compared with control group; ^#^ *P* < 0.05 compared with cervical cancer + anti PD-L1 group; ^△^ *P* < 0.05 compared with CIN + anti-PD-L1 group.

To investigate the effect of PD-L1 on lymphocyte proliferation and the secretion of IL-10 and TGF-*β*, PD-L1 was blocked using anti-PD-L1 and stimulated by ConA. MTT assay showed that lymphocyte proliferation in cervical cancer group and CIN group was significantly lower than control group (*P* < 0.05). However, blockade of PD-L1 in cervical cancer group significantly enhanced lymphocyte proliferation (*P* < 0.05) (Figure 6(a)). CBA assay showed that the levels of IL-10 and TGF-*β* in lymphocyte culture supernatants from cervical cancer group and CIN group were significantly higher than those from control (*P* < 0.05). After addition of anti-PD-L1 antibody, the levels of IL-10 and TGF-*β* were significantly reduced (*P* < 0.05) but still significantly higher than those in control (*P* < 0.05) (Figures 6(b) and 6(c)). These results suggest that PD-1/PD-L1 pathway inhibits lymphocyte proliferation but enhances the secretion of IL-10 and TGF-*β* in vitro.

## 4. Discussion

PD-1 is an important negative immune regulatory molecule belonging to CD28/CTLA-4 family. The extracellular IgV-like domain of PD-1 contains four important N-linked glycosylation sites that can bind with its ligands PD-L1 or PD-L2. It plays a regulatory role in immune responses by conducting inhibitory signals via immunoreceptor tyrosine-based inhibition motif [8]. Konishi et al. have detected high expression of PD-1 in non-small cell lung cancer T lymphocytes [9]. Yang et al. discover that upregulated PD-1/PD-L1 expression is positively correlated with the progression and degree of CIN induced by HPV infection [10]. In the present study, we find that the PD-1/PD-L1 pathway is upregulated in CIN patients, and the levels of TGF-*β* and IL-10 are elevated, consistent with the results by Yang et al. Further study shows that PD-1 expression on the surface of CD4+T and CD8+T cells, PD-L1 expression on DC surface, and mRNA expression of PD-1 and PD-L1 are significantly enhanced in cervical cancer patients, being higher than that in CIN group. As the development of cervical lesions, the level of PD-1 is elevated, suggesting that PD-1/PD-L1 expression is closely related with the development of cervical lesions. PD-1/PD-L1 signaling pathway may facilitate the occurrence of cervical cancer by inhibiting the immune functions of T cells.

Hamanishi et al. report that PD-1 expression and CD8+T infiltration degree in ovarian cancer are negatively correlated with the prognosis of patients [11]. Shi et al. find that expression of PD-1 is significantly enhanced in tumor and its adjacent tissues, promoting the apoptosis of CD8+T cells that are in contact with liver cancer cells [12]. Another study indicates that PD-1 inhibits antitumor immunity at CD8+T cell effector phase [13]. A study claims that activation of PD-1/PD-L1 signaling pathway leads to the formation of immunosuppressive tumor microenvironment that helps tumor cells evade immune surveillance and destruction, and blocking of PD-1/PD-L1 signaling pathway reverses tumor immune microenvironment [14]. Another study shows that tumor cells evade T cell recognition by upregulating PD-L1 expression [15]. PD-1 and PD-L1 inhibit TCR-mediated stop signals and lead to T cell tolerance.

We discover that the increase of PD-1+CD8+T is correlated with tumor differentiation, lymph node metastasis, and tumor metastasis. High expression of PD-1 in CD8+T cells may inhibit the scavenging effect of CD8+T cells on tumor cells. Therefore, tumor proliferation is promoted and invasion and metastasis occur. Heeren et al. claim that lymph node metastasis of cervical cancer is correlated with the increase of PD-1^+^T cells and PD-L1^+^APCs, leading to the development and metastasis of cervical cancer [16].

Our previous studies demonstrate that Treg cells are increased as the aggravation of cervical cancer lesions. We speculate that Treg cells play important roles in immune evasion of tumor cells [17, 18]. Another report shows that activated Treg cells express PD-1 and exert effector mechanism via PD-1/PD-L1 pathway [19]. Kitazawa et al. find that blocking of PD-1/PD-L1 pathway inhibits inhibitory function of Treg cells and restores the proliferation of CD4+CD25-T cells [20]. PD-1 signal interferes with the secretion of multiple cytokines [21]. Inflammatory mediators such as IL-1 and IL-2 induce the expression of PD-L1 that exerts feedback inhibition effect [22]. TGF-*β* and IL-10 can also help antigen-presenting cells express PD-L1 via STAT-3 to exert negative regulation effect [23]. After binding of PD-L1 with PD-1 on activated T cells, the antitumor effect of T cells is inhibited [24]. The present study shows that the concentrations of TGF-*β* and IL-10 are elevated while that of IFN-*γ* is reduced in cervical cancer. Correlation analysis shows that PD-1 expression in Treg cells is positively correlated with TGF-*β* and IL-10 but negatively correlated with IFN-*γ*, suggesting that PD-1/PD-L1 participates in the inhibition mechanism of Treg cells via TGF-*β* and IL-10. It is speculated that PD-1/PD-L1 signaling pathway has potential effect in regulating the immune suppression function of Treg cells in cervical cancer patients, leading to the malignancy of the disease.

The study also shows that PD-L1 on DC cells is capable of inhibiting T cell activation and lysing tumor cells or sometimes leads to the apoptosis of tumor-specific T cells. Blocking of PD-L1 enhances the efficacy of immune therapy. A study shows that PD-L1 positive P815 tumor cells resist immune therapy, but blocking of PD-L1 restores the effect of anti-CD137 monoclonal antibody therapy [25]. PD-L1 is also expressed on tumor-related antigen-presenting cells and regulates T cell-mediated antitumor effect via PD-1/PD-L1 signaling pathway [26]. By blocking PD-1/PD-L1 signaling pathway, the proliferation of CD8+T cells in tumor microenvironment can be restored, and more effector cytokines and cytotoxic enzymes can be produced. In addition, tumor antigen-specific CD8+T cells can secrete more IFN-*γ*. These facts support the speculation that PD-1/PD-L1 pathway blocking therapy can be used to enhance antitumor immunity [27, 28]. Using PD-L1 antibody to block PD-1/PD-L1 pathway, the present study discovers that lymphocyte proliferation is promoted and secretion of IL-10 and TGF-*β* is reduced after blocking of PD-L1. Yang et al. report that PD-1/PD-L1 pathway inhibits cellular immunity, leading to the occurrence and development of CIN [10]. Our results demonstrate that PD-1/PD-L pathway inhibits T lymphocyte proliferation, promotes the secretion of TGF-*β* and IL-10, and results in the proliferation of differentiation of Tregs in cervical cancer microenvironment, finally leading to the escape of tumor cells from immune system. PD-L1 expression in tumor cells enhances the apoptosis of antigen-specific T cells. Therefore, blocking of PD-L1 reduces T cell apoptosis. Recent studies show that depletion of tumor-infiltrating lymphocytes in tumor microenvironment is correlated with PD-L1 secreted by tumor cells or tumor-derived myeloid cells. Blocking of PD-1/PD-L1 signaling pathway enhances the function of effector CD8+T cells, inhibits the functions of Treg cells and myeloid-derived suppressor cells, and increases the antitumor immunity effect [29–31]. Therefore, blocking of PD-1/PD-L1 pathway in the treatment of cervical cancer may be helpful in restoring T cell proliferation ability in tumor microenvironment, generating more effector cell factors, and enhancing immune effect against cervical cancer.

In summary, the present study finds that upregulation of PD-1 on T cells and PD-L1 on DCs in cervical cancer may inhibit T cell proliferation and function in tumor microenvironment, leading to immune suppression that facilitates the growth and metastasis of tumors. The results of relevance between changes of PD-1/PD-L1 and Treg cells demonstrate that PD-1/PD-L1 signaling pathway may exert its immune suppression effect via Treg cells and its factors TGF-*β* and IL-10, leading to the occurrence of cervical cancer. The present study provides a new idea that blocking of PD-1/PD-L1 signaling pathways using PD-1 or PD-L1 antibodies may provide a therapeutic method in the immune treatment of cervical cancer.

## Figures and Tables

**Table 1:** Clinical characteristics of patients with cervical cancer.

Characteristics	Categories	Number (percentage, %) of patients (*N* = 78)
Age (years)	<45	60 (76)
	>45	18 (24)

FIGO stage	I	31 (39)
	II	34 (43)
	III	8 (10)
	IV	5 (6)

Histological types	SCC	66 (85)
	ADC/ADSC	12 (15)

Tumor differentiation	Well	11 (14)
	Moderate	28 (36)
	Poor	39 (50)

Lymph node metastases	Positive	26 (33)
	Negative	52 (67)

Tumor size (cm)	<4	43 (55)
	≥4	35 (45)

Tumor invasion depth (mm)	<15	41 (53)
	≥15	37 (47)

Tumor metastasis	Yes	28 (36)
	No	47 (60)
	Unknown	3 (4)

**Table 2:** Clinical characteristics of patients with cervical intraepithelial neoplasia (CIN).

Number of patients	53

Age (years)	Median
	Range

Clinical stages	CIN I
	CIN II
	CIN III
